# Effect of Microenvironment on Differentiation of Human Umbilical Cord Mesenchymal Stem Cells into Hepatocytes* In Vitro* and* In Vivo*

**DOI:** 10.1155/2016/8916534

**Published:** 2016-02-10

**Authors:** Gai Xue, Xiaolei Han, Xin Ma, Honghai Wu, Yabin Qin, Jianfang Liu, Yuqin Hu, Yang Hong, Yanning Hou

**Affiliations:** ^1^Graduate School, Hebei Medical University, Shijiazhuang, Hebei 050017, China; ^2^Department of Pharmacy, Bethune International Peace Hospital, Shijiazhuang, Hebei 050082, China

## Abstract

Human umbilical cord-derived mesenchymal stem cells (hUCMSCs) are considered to be an ideal cell source for cell therapy of many diseases. The aim of this study was to investigate the contribution of the microenvironment to the hepatic differentiation potential of hUCMSCs* in vitro* and* in vivo* and to explore their therapeutic use in acute liver injury in rats. We established a new model to simulate the liver tissue microenvironment* in vivo* using liver homogenate supernatant (LHS)* in vitro*. This induced environment could drive hUCMSCs to differentiate into hepatocyte-like cells within 7 days. The differentiated cells expressed hepatocyte-specific markers and demonstrated hepatocellular functions. We also injected hUCMSCs into rats with CCl_4_-induced acute hepatic injury. The hUCMSCs were detected in the livers of recipient rats and expressed the human hepatocyte-specific markers, suggesting that hUCMSCs could differentiate into hepatocyte-like cells* in vivo* in the liver tissue microenvironment. Levels of biochemistry markers improved significantly after transplantation of hUCMSCs compared with the nontransplantation group (*P* < 0.05). In conclusion, this study demonstrated that the liver tissue microenvironment may contribute to the differentiation of hUCMSCs into hepatocytes both* in vitro* and* in vivo*.

## 1. Introduction

Acute liver injury is a frequent response to a variety of acute injuries [1]. Many patients with acute liver injury require orthotopic liver transplantation because of the failure of pharmacological treatments [2]. However, liver transplantation is limited by a shortage of transplantable donor organs, and stem cell transplantation has been proposed as a viable alternative to organ transplantation in patients with acute liver injury. Stem cell therapy and its associated mechanisms have been the subject of extensive recent research [3–5]. Mesenchymal stem cells (MSCs) are excellent candidates for cell therapy because of their capacity for self-renewal, multipotency, low immunogenicity, and availability. Several studies have reported on the isolation of MSCs from various sources, including bone marrow, placenta, amniotic fluid, adipose tissue, and umbilical cord blood [6, 7]. Among these sources, MSCs isolated from umbilical cord (UCMSCs) can be obtained by a noninvasive procedure and can be easily cultured, making them potentially superior candidates for cell transplantation compared with MSCs from other sources [8].

A stem cell niche is defined as the microenvironment surrounding stem cells and is composed of adjacent cells, adhesive molecules, and extracellular matrix [9]. The microenvironment not only provides nutrients for the stem cells but also maintains their characteristics of quiescence and self-renewal. Changes in the microenvironment can activate stem cells to undergo proliferation or differentiation into various types of functional cells. The microenvironment created by the culture medium can maintain the continuous proliferation of isolated stem cells* in vitro*. After MSC transplantation into a recipient, they are then able to differentiate into mature tissue cells consistent with the surrounding environment [10]. However, it is unclear if the precise mechanism of differentiation involves the combined actions of a variety of cytokines or the activation of signaling pathways within stem cells as a result of cell contact. MSCs differentiated* in vitro* as a result of cytokine induction often lack the functions of tissue cells [11, 12]. Our previous studies found that the differentiation time* in vivo* tended to be shorter and the differentiation efficiency tended to be higher compared to* in vitro*, indicating the need for other factors to accelerate the differentiation process, in addition to cytokines. However, there is currently no appropriate* in vitro* model simulating the stem cell microenvironment. Recent studies have shown that tissue extracts can induce stem cell differentiation into functional cells* in vitro* [13, 14] but the ability of liver tissue extract to induce UCMSC differentiation into hepatocytes remains unknown.

We previously isolated hUCMSCs that expressed MSC markers and demonstrated the capacities for osteogenic and adipogenic differentiation* in vitro* [13]. In this study, we investigated the effect of the microenvironment on hUCMSC differentiation using liver homogenate supernatants (LHS) to simulate the liver tissue microenvironment. The results of this study will further our understanding of the role of the microenvironment and provide important information relevant to the clinical application of hUCMSCs.

## 2. Materials and Methods

### 2.1. Isolation, Culture, and Identification of hUCMSCs

#### 2.1.1. Isolation and Culture of hUCMSCs

The present study was approved by the Research Ethics Committee at Bethune International Peace Hospital of People's Liberation Army. Umbilical cords from full-term normal and cesarean deliveries were obtained from the department of gynaecology and obstetrics, with the mothers' consent. hUCMSCs were isolated and identified by flow cytometry, as described previously [13]. Cell pellets were suspended in expansion medium containing Dulbecco's Modified Eagle's Medium/F12 (DMEM/F12) (HyClone, Rockville, MD, USA) with 10% fetal bovine serum (FBS) (HyClone), 100 U/mL penicillin, and 100 mg/mL streptomycin. Plated cells were cultured in expansion medium at 37°C and 5% CO_2_ in a fully humidified atmosphere.

#### 2.1.2. Flow Cytometry Phenotyping of hUCMSCs

The phenotype of the hUCMSCs was evaluated by flow cytometry (EPICS-XL4, Beckman Coulter, Inc., 250 S Kraemer Boulevard Brea, CA, USA). Native third- to fifth-passage hUCMSCs were trypsinized and suspended in phosphate-buffered saline (PBS) at a concentration of 1 × 10^7^ cells/mL. The following mouse anti-human monoclonal antibodies were used: CD73-phycoerythrin (PE) (BD Pharmingen, Franklin Lack, NJ, USA); CD90-fluorescein isothiocyanate (FITC) and CD31-PE (BioLegend, San Diego, CA, USA); and CD105-PE, CD29-FITC, CD45-PC5, CD34-PE, and HLA-DR-FITC (BD Biosciences, CA, USA). FITC- as well as PE-labeled mouse immunoglobulin G was used as a negative control. The cells and antibodies were incubated at 4°C for 30 min and washed three times with PBS. Labeled cells were assayed by flow cytometry and analyzed with Expo32 software.

#### 2.1.3. Osteogenic and Adipogenic Differentiations of hUCMSCs

hUCMSCs in passage 3 were cultured in DMEM/F12 (HyClone) medium with 10% FBS, containing either osteogenic (0.01 *μ*M dexamethasone, 10 mM *β*-glycerophosphate, 50 mg/L ascorbate phosphate, 100 U/mL penicillin, and 100 U/mL streptomycin) or adipogenic (1 *μ*M dexamethasone, 50 mg/L ascorbate phosphate, 100 mg/L 3-isobutyl-1-methylxanthine, 100 U/mL penicillin, and 100 U/mL streptomycin) reagents (all from Sigma, Saint Louis, MO, USA). hUCMSCs cultured in regular medium were used as a control. Osteogenic differentiation was assessed after 2 weeks by examining calcified base sediment with Von Kossa staining, and adipogenic differentiation was examined via intracellular accumulation of lipid visualized using Oil-Red-O staining, under an inverted microscope.

### 2.2. Preparation of Induction Medium-LHS

Livers of Sprague-Dawley rats were exposed after anesthesia and then perfused for 20 min using 4°C physiological saline via the hepatic portal vein. After perfusion, hepatic tissue was placed on ice, the tegument and ligaments were removed, and the wet weight was measured. Subsequently, 150 mg of liver with 1 mL of DMEM/F12 was then homogenized 40 times in an ice bath using a manual glass homogenizer. The liver homogenate was centrifuged at 15,000 ×g for 30 min at 4°C. The supernatant was then filtered through a 0.22-*μ*m filter and incubated at 37°C in 5% CO_2_ for 24 h. Supernatants were collected and 10% FBS was added for further investigations.

### 2.3. Hepatocyte Differentiation of hUCMSCs* In Vitro*

Passage 3 hUCMSCs were divided into control (cultured in basic culture medium; 0 d group) and LHS (cultured in LHS for 3, 5, and 7 days; 3 d, 5 d, 7 d groups) groups. Positive (QSG-7701 human liver cell line; PC group) and negative (LHS without cells to control for interference from proteins in LHS; NC group) control groups were also set up. Undifferentiated cells were harvested after 4 days of culture at 80% confluence. For differentiation assays, hUCMSCs at 80% confluence were treated with LHS for 3, 5, and 7 days, respectively. Cultures were maintained by changing the medium every 2 days. Protein levels of the hepatocyte markers, *α*-fetoprotein (AFP), cytokeratin 18 (CK18), and tryptophan 2,3-dioxygenase (TPH2) were analyzed at different time points by western blotting, and the functions of hepatocyte-like cells, including CYP3A enzymatic activity, urea production, and albumin (ALB) externalization, were detected.

### 2.4. Characterization and Functional Detection of hUCMSCs after Induction

#### 2.4.1. Protein Extraction and Western Blot Analysis

Western blot analysis was performed to quantify the expression of human liver cell-specific marker proteins in induced hUCMSCs* in vitro*. Briefly, cells were rinsed twice with PBS (4°C), scraped with a cell scraper, and collected into 1.5-mL Eppendorf tubes in radioimmunoprecipitation assay (RIPA) cell-disruption buffer (1 mL RIPA plus 10 *μ*L phenylmethanesulfonyl fluoride) and kept in an ice bath for 30 min and then centrifuged at 12,000 ×g for 10 mins at 4°C. The supernatant contained cell total protein. The protein concentration of the cells was determined using a bicinchoninic acid protein assay kit (GENEray Biotechnology, Ltd., Shanghai, China). Twenty micrograms of tissue lysate was added to a 10% sodium dodecyl sulfate-polyacrylamide gel, transferred to a polyvinylidene difluoride membrane, and blocked with 5% dried skim milk. Anti-AFP, CK18 monoclonal antibody, anti-TPH2 polyclonal antibody (consistent with the antibodies used for immunohistochemistry), and *β*-actin polyclonal antibody (Beijing ComWin Biotech Co., Ltd., Beijing, China) were added at 1 : 100, 1 : 10,000, 1 : 100, and 1 : 3,000 dilutions, respectively, overnight at 4°C, followed by incubation with horseradish peroxidase-conjugated secondary antibody (1 : 2,000, Beijing ComWin Biotech Co., Ltd., Beijing, China). Immunodetected proteins were visualized using the enhanced chemiluminescence (ECL) method with a low-background ECL kit (Beijing ComWin Biotech Co., Ltd.). Protein bands were quantified using image analysis software (ImageJ Quantity One, Bio-Rad, Hercules, CA, USA). Protein expression was determined as the optical density (562 nm) of each sample divided by that for *β*-actin.

#### 2.4.2. Determination of CYP3A Enzymatic Activity

Midazolam was used as effective substrate for CYP3A, and a reduction in midazolam concentration indicated an increase in CYP3A enzymatic activity. Medium containing midazolam was added to induced cells for 2 h at 37°C and the medium was then collected. Midazolam concentrations were determined using an Agilent HP-1100 liquid chromatography mass spectrometer with an electrospray ionization source (Agilent, Santa Clara, CA, USA). Chromatographic separation was achieved on a Gemini C18 analytical column (50 mm × 2.0 mm, 5 *μ*m, Phenomenex, Torrance, CA, USA) with C18 guard column (3.0 mm × 4.0 mm, 5 *μ*m, Phenomenex). A standard stock solution of midazolam was dissolved in water to obtain an exact final concentration of 100 *μ*g/mL and stored at 4°C. The target calibration range was 10–500 ng/mL (limit of detection 10 ng/mL). Diazepam was used as an internal standard at a concentration of 100 ng/mL. The flow rate was set at 0.2 mL/min. The mobile phase consisted of acetonitrile : water (60/40, v/v, containing 0.2% glacial acetic acid). Midazolam was ionized under positive ionization conditions and 10 mL was analyzed on a column. The midazolam (*m/z*) 326 ion was selected.

#### 2.4.3. Determination of Urea and ALB

Urea production by hepatocyte-like cells was determined using a blood urea nitrogen test kit (Nanjing Jiancheng Bioengineering Institute, Nanjing, Jiangsu, China), and ALB was determined by enzyme-linked immunosorbent assay (ELISA) (ImmunoWay Biotechnology Company, Newark, DE, USA), according to the manufacturers' instructions.

### 2.5. Animal Model of CCl_4_-Induced Acute Liver Injury and Transplantation of hUCMSCs

Male Sprague-Dawley rats (body weight 180–220 g) were purchased from the Laboratory Animal Center, Hebei Medical University, China. All experimental procedures were carried out in accordance with Chinese legislation regarding experimental animals, and these experiments were approved by the Animal Experiment Ethics Committee at Bethune International Peace Hospital of PLA. Rats were given a single intraperitoneal injection of 50% CCl_4_ (Aladdin Chemical Co., Ltd, Nanqiao Town, Shanghai, China) at a dose of 3 mL/kg in vegetable oil before transplantation. Cells marked with PKH26 (red fluorescent cell linker kit for general cell membrane labeling to allow the transplanted cells to be tracked, Sigma) were transplanted 24 h after administration of CCl_4_ via tail vein injection. Rats were separated into five groups of 18 rats each: naive group (no treatment), model group (injected with CCl_4_ only), treatment group (CCl_4_-treatment plus undifferentiated hUCMSCs (1 × 10^6^ cells) in 500 *μ*L of normal saline (NS) injected into their tail veins), vehicle group (500 *μ*L of NS injected into their tail veins), and control group (normal rats injected with 1 × 10^6^ undifferentiated hUCMSCs in 500 *μ*L of normal saline (NS) into their tail veins). Liver and blood samples were collected from each group and prepared for further analysis at 3 h, 2 days, and 7 days after treatment.

### 2.6. Fluorescent Labeling of hUCMSCs* In Vitro* and Distribution in CCl_4_-Injured and Normal Rat Liver* In Vivo*

hUCMSCs were washed three times with PBS and labeled with PKH26 (Sigma) following the manufacturer's instructions. After labeling, cell proliferation was examined by drawing a growth curve, and the effect of cell propagation on PKH26 labeling was observed. Cell nuclei were stained with Hoechst33342 (Solarbio, Beijing, China) before observing under a fluorescence microscope (BX41, Olympus, Tokyo, Japan).

Freshly dissected livers were immersed in optimal cutting temperature compound and frozen in liquid nitrogen. Six-micrometer sections were cut using a freezing microtome (CM1900, Leica, Heidelberg, Germany). For fluorescent immunohistochemistry, liver slides were incubated with diluted primary antibody against anti-rat EMR1 (1 : 100, Biosynthesis Biotechnology, Beijing, China) and then observed directly using a fluorescence microscope (BX41, Olympus, Tokyo, Japan).

### 2.7. Immunohistochemistry

Rat liver samples were fixed in 4% paraformaldehyde solution at room temperature overnight. Liver slides were then incubated with diluted primary antibodies against anti-human CK18 (1 : 100, Abcam, Cambridge Science Park, Cambridge, UK), human AFP (1 : 50), human TPH2 (1 : 20), and human ALB (1 : 50) (Cell Signaling Technology, Inc., Danvers, MA, USA), followed by biotin-conjugated secondary antibody. Immunoreactivity was visualized by developing sections with diaminobenzidine and nuclear counterstaining with hematoxylin-eosin (HE). For each staining, control sections were incubated with 1% bovine serum albumin in PBS instead of the primary antibodies.

### 2.8. Assessment of Liver Functions

Blood samples were obtained 3 h and 2 and 7 days after hUCMSC transplantation and centrifuged at 1000 ×g for 15 mins, and the serum was collected. Serum samples were tested for alanine aminotransferase (ALT), aspartate aminotransferase (AST), malondialdehyde (MDA), and total bilirubin (TBIL) using ALT/GPT microplate test kit, AST/GOT microplate test kit, MDA test kit, and TBIL test kit, respectively (Nanjing Jiancheng Bioengineering Institute).

### 2.9. Statistical Analysis

Data were expressed as mean ± standard deviation (SD). Comparisons were made using one-way analysis of variance and Dunnett's *t*-tests. Statistical analyses were performed using SPSS13.0 statistical software (SPSS, Chicago, IL, USA), and *P* < 0.05 was considered to indicate statistical significance.

## 3. Results

### 3.1. Characterization of hUCMSCs

**Figure 1:**
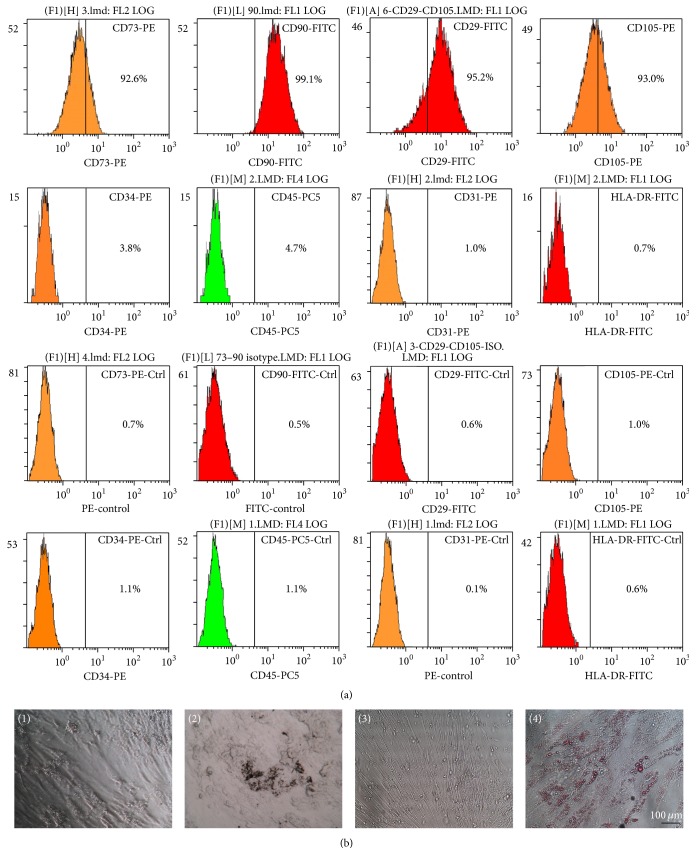
Characterization of hUCMSCs. (a) Marker expression in hUCMSCs at passage 3, revealed by flow cytometry. (b) Determination of differentiation of hUCMSCs; ((1) and (3)) control groups; (2) osteogenic differentiation of hUCMSCs shown by calcium deposits revealed by Von Kossa staining (×400); (4) adipogenic differentiation of hUCMSCs stained by Oil-Red-O (×400).

**Figure 2:**
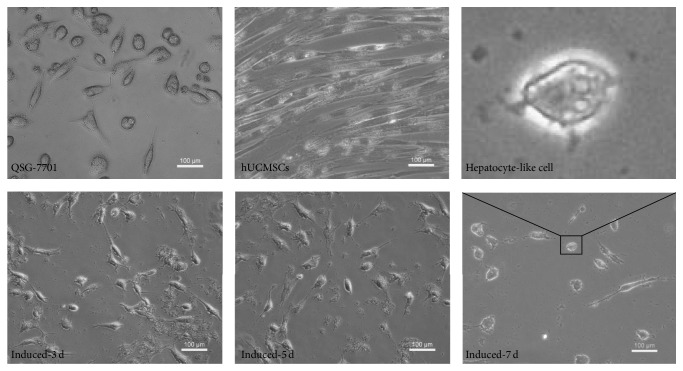
Morphologic changes in hUCMSCs after induction by LHS.

**Figure 3:**
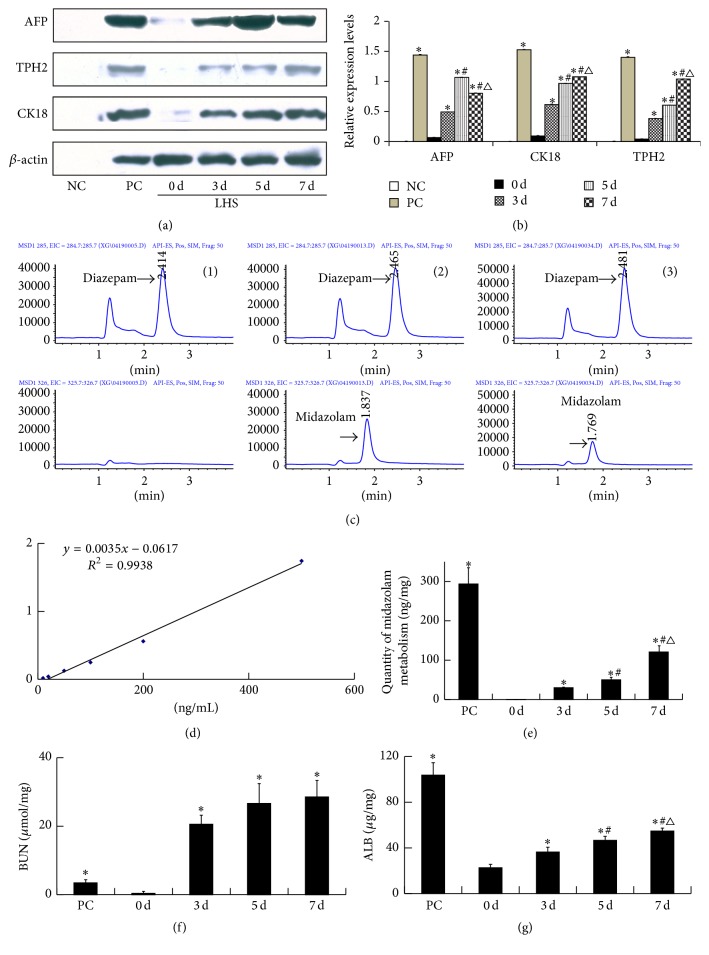
Determination of hepatocyte markers and function after differentiation* in vitro*. ((a) and (b)) Determination of hepatocyte markers by western blotting. (c) Typical high-performance liquid chromatography/mass spectrometry chromatograms of midazolam and diazepam (internal standard) in culture media samples. (1) Blank culture medium + diazepam; (2) blank culture medium + midazolam (500 ng/mL) + diazepam; (3) sample culture medium after 2 h incubation with hUCMSCs induced by LHS. (d) Linear curve for midazolam. (e) Effects of LHS on CYP3A enzyme activity in hUCMSCs after 3, 5, and 7 days. (f) Effects of LHS on blood urea nitrogen production in hUCMSCs after 3, 5, and 7 days. (g) Effects of LHS on ALB externalization in hUCMSCs after 3, 5, and 7 days (mean ± SD, *n* = 6, ^*∗*^ *P* < 0.01 compared with 0 d, ^#^ *P* < 0.05 compared with 3 d, and ^△^ *P* < 0.05 compared with 5 d). hUCMSC, human umbilical cord-derived mesenchymal stem cell; NC, negative control group; PC, positive control group of QSG-7701 human hepatic cell line; 0 d, undifferentiated hUCMSCs; 3 d, 5 d, 7 d, 3 d, 5 d, and 7 days after induction by LSH, respectively; AFP, *α*-fetoprotein; CK18, cytokeratin 18; TPH2, tryptophan 2,3-dioxygenase; LHS, liver homogenate supernatant.

**Figure 4:**
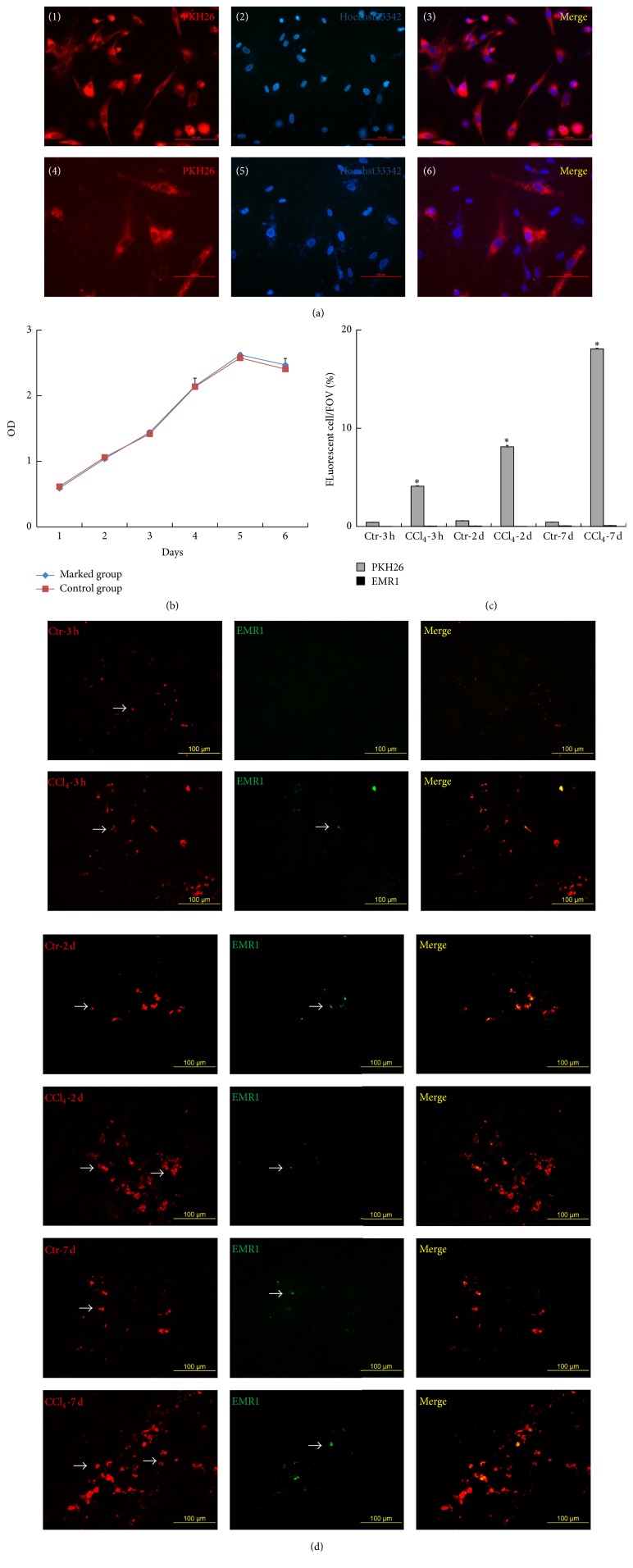
Fluorescence imaging of PKH26^+^ cells* in vitro* and* in vivo*. (a) Duration of fluorescence after hUCMSC staining by PKH26* in vitro*. (1) Nonpassage hUCMSCs marked with PKH26. (4) Four passages of hUCMSCs after PKH26 staining. ((2) and (5)) Cell nuclei stained with Hoechst33342 in the same field of vision as (1) and (4). (3, 6) Merged images. (b) Effect of PKH26 on hUCMSC growth curve. (c) Quantification of PKH26^+^ and EMR1^+^ cells in liver. (d) EMR1 immunofluorescence in frozen rat liver sections after transplantation of PKH26 marked hUCMSCs. Positive cells indicated by arrow (mean ± SD, *n* = 6, and ^*∗*^ *P* < 0.01 compared with control). Bar = 100 *μ*m. FOV, field of view.

Fibroblast-like cells began to grow out from the umbilical cord pellets between the 10th and 14th day of primary culture and reached 80%–90% confluence in a whirlpool or radiating manner after 7–10 days. The cells expressed high levels of the MSC-specific surface markers CD73, CD90, CD29, and CD105, as demonstrated by flow cytometry, but lacked expression of the hematopoietic and endothelial cell-specific markers CD34, CD45, and CD31 as well as human leukocyte antigen (HLA) class II (HLA-DR) (Figure 1(a)). These results confirmed these cells as MSCs, rather than hematopoietic or endothelial cells. Control hUCMSC cultures showed no differentiation (Figure 1(b), (1) and (3)). Nodules of calcium mineralization were formed, as revealed by Von Kossa stain after osteogenic induction (Figure 1(b), (2)), and numerous lipid droplets were observed in hUCMSCs with Oil-Red-O staining, after incubation with adipogenic supplement for 14 days (Figure 1(b), (4)). These results showed that the cells displayed characteristics of MSCs in terms of character and differentiation ability.

### 3.2. *In Vitro* Differentiation of hUCMSCs into Hepatocyte-Like Cells

After induction, spindle-shaped stem cells began to lose their sharp edges and shrink progressively, before turning into triangular, polygonal, and irregular hepatocyte-like cells (Figure 2).

Western blotting revealed no protein in negative control cells and protein expression of liver-cell-specific markers (AFP, CK18, and TPH2) in induced cells. Compared with 0 d group, protein levels of AFP, CK18, and TPH2 were significantly increased after induction with LHS at different time points (*P* < 0.01). AFP expression peaked at 5 days and then declined, while expression levels of CK18 and TPH2 increased with prolonged induction (*P* < 0.05) (Figures 3(a) and 3(b)). These data suggested that hUCMSCs could express hepatocyte-specific markers after induction with LHS.

### 3.3. *In Vitro* Differentiated hUCMSCs Exhibit Hepatocyte Functions

The retention times of midazolam and diazepam were 1.8 and 2.4 min, respectively. Under experimental conditions, DMEM/F12 medium did not interfere with the determination of midazolam concentration (Figure 3(c)). The coefficients of variation were <15% and the determination coefficient (*r*) was 0.9969 (Figure 3(d)).

hUCMSCs showed very little midazolam metabolism (0 d metabolism, 0.026 ng/mg), while metabolism in the induction group at 3, 5, and 7 days was increased to 30.14 ± 1.19, 50.20 ± 6.24, and 120.85 ± 15.52 ng/mg, respectively. Compared with 0 d group, midazolam metabolism was significantly increased in the LHS group (*P* < 0.01) in a time-dependent manner, indicating that LHS induced CYP3A activity in an induction-time-dependent manner (Figure 3(e)). Stem cells secreted very little urea (0 d secretion, 0.40 *μ*mol/mg), but urea secretion increased >66-fold after induction, to 20.59 ± 2.61, 26.64 ± 5.79, and 28.55 ± 4.80 *μ*mol/mg at 3, 5, and 7 days, respectively. Compared with 0 d group, urea secretion was significantly increased at 3, 5, and 7 days (*P* < 0.01) (Figure 3(f)). Stem cells have the ability to secrete ALB (0 d group, 22.66 ± 2.99 *μ*g/mg), and ALB secretion was significantly increased to 36.40 ± 3.53, 46.66 ± 2.50, and 54.82 ± 4.28 *μ*g/mg at 3, 5, and 7 days of induction, respectively (*P* < 0.01) (Figure 3(g)). In summary, CYP3A enzyme activity and ALB and urea secretion were significantly increased by LHS induction.

### 3.4. *In Vivo* Tracking of PKH26-Labeled hUCMSCs after Transplantation into Rats

**Figure 5:**
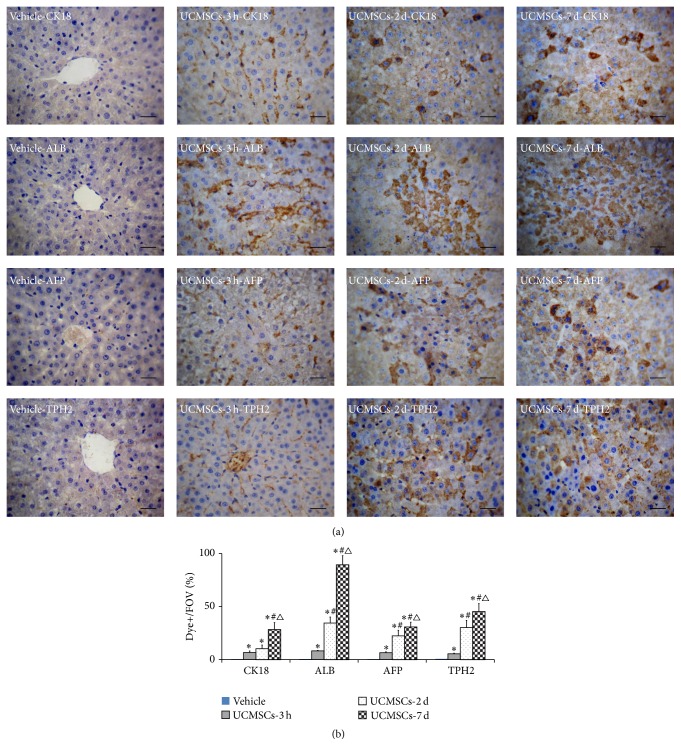
Immunohistochemical determination of human hepatocyte markers in rat liver after transplantation of hUCMSCs. (a) Expression of CK18, ALB, AFP, and TPH2 in liver after transplantation of hUCMSCs into acute CCl_4_-treated rats. (b) Quantification of human hepatocyte-specific marker positive cells (mean ± SD, *n* = 6, ^*∗*^ *P* < 0.01 compared with vehicle group, ^#^ *P* < 0.05 compared with 3 h group, and ^△^ *P* < 0.05 compared with 2 d group). Bar = 100 *μ*m.

**Figure 6:**
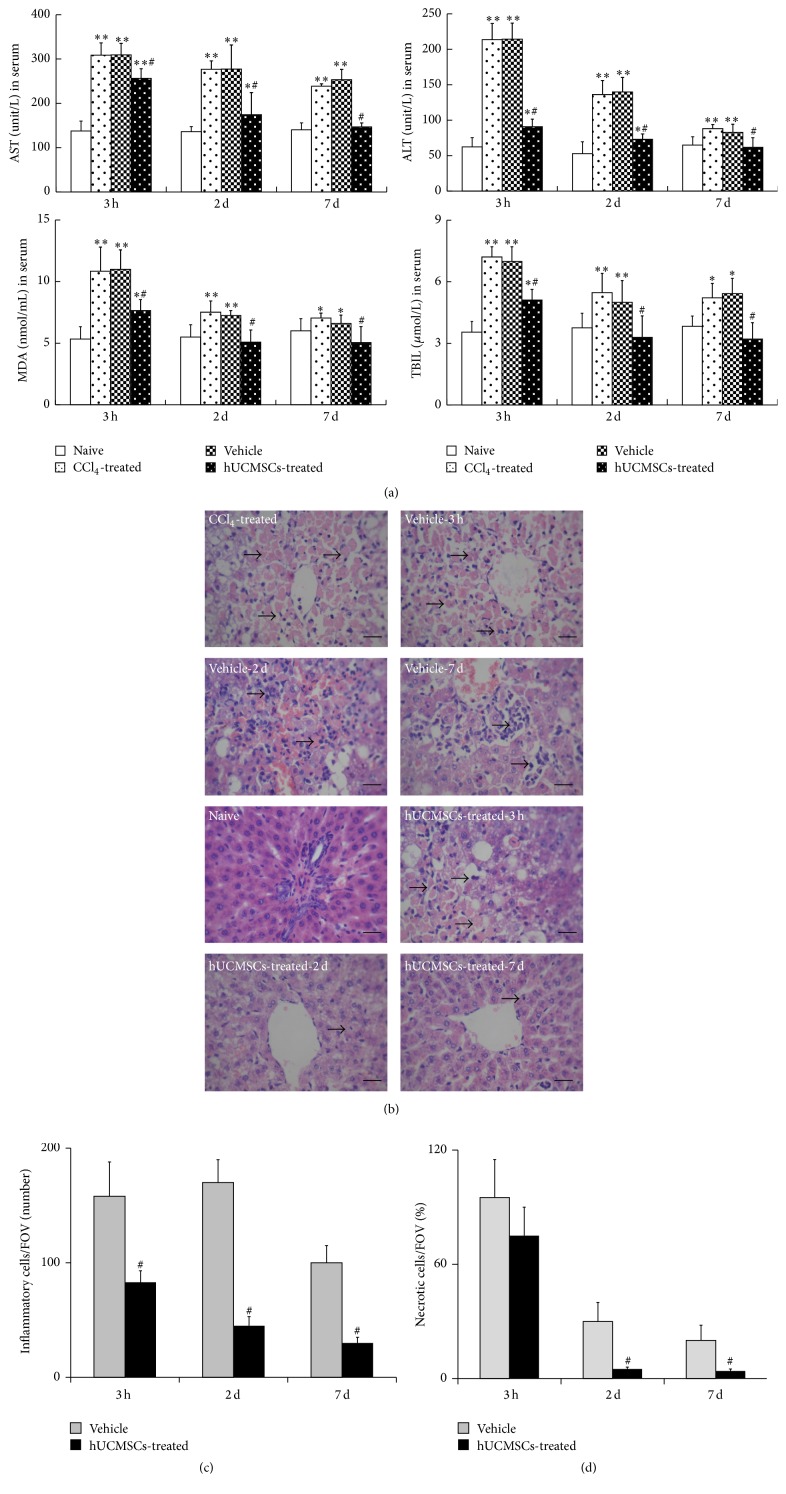
Effects of hUCMSC transplantation on recovery of acute CCl_4_-induced liver injury. (a) Effects of hUCMSC transplantation on liver function. (b) Histopathological recovery of liver in CCl_4_-treated rats transplanted with hUCMSCs. Inflamed, denatured, and necrotic cells are indicated by arrows. (mean ± SD, *n* = 6, ^*∗*^ *P* < 0.05, and ^*∗∗*^ *P* < 0.01 compared with naïve group; ^#^ *P* < 0.05 compared with vehicle). Bar = 100 *μ*m. AST, aspartate aminotransferase; ALT, alanine aminotransferase; MDA, malondialdehyde; TBIL, total bilirubin.

PKH26 marked 100% of hUCMSCs* in vitro* (Figure 4(a), (1), (2), and (3)) and the fluorescence could be maintained for 20 days after labeling* in vitro* (Figure 4(a), (4), (5), and (6)). PKH26 had no significant influence on hUCMSC proliferation (Figure 4(b)). PKH26 was thus an ideal fluorescent dye for labeling hUCMSCs.

hUCMSCs labeled with PKH26 were injected into rats via the tail vein. Three randomly selected views in each liver section and six sections per group were evaluated. The numbers of fluorescent cells in the liver increased after 3 h, 2 days, and 7 days compared with the control group (Figure 4(d)). Liver damage might be one of the factors associated with the enrichment of hUCMSCs in the liver. We therefore examined the macrophage marker EMR1 by immunofluorescence in the frozen sections simultaneously. Although EMR1 was expressed in liver, its expression was low compared with PKH26^+^ cells. These results showed that most (86.32%–99.76%) of the PKH26^+^ cells were active cells, rather than indicating PKH26 that had been taken up by macrophages. These experiments thus revealed the homing and location of hUCMSCs in the injured liver.

### 3.5. Human UCMSCs Differentiated into Human Hepatocytes Expressing Specific Markers in Injured Rat Livers after Transplantation

We confirmed that hUCMSCs differentiated into hepatocytes by determining the expression of the human hepatocyte-specific markers AFP, CK18, ALB, and TPH2 by immunohistochemistry. CCl_4_-treated rat liver not injected with hUCMSCs was used as a vehicle control. Human hepatocyte-specific markers were detected in the liver 3 h after injection, but the cells did not resemble hepatocyte-like cells. However, by 2 and 7 days after injection, hepatocyte-like cells expressing CK18, AFP, ALB, and TPH2 were found. The levels of hepatocyte-specific markers increased in a time-dependent manner. No AFP, CK18, ALB, or TPH2 expression was observed in livers in the vehicle control group (Figure 5). These results confirmed that hUCMSCs could secrete hepatocyte-specific markers at 3 h and continued to differentiate into hepatocyte-like cells in injured liver tissue.

### 3.6. hUCMSCs Alleviated CCl_4_-Induced Rat Liver Injury

To determine if hUCMSCs had the therapeutic ability to repair injured liver, we observed the liver morphology and pathological structure in all groups by HE staining. Pathological examination of HE-stained sections revealed inflammation (deep blue staining, indicated by arrows, Figure 6(b)), cellular degeneration, and massive necrosis (pink homogeneous material, indicated by arrows, Figure 6(b)) in the CCl_4_-treated group compared with naive liver tissue, as well as suppression of hepatocyte necrosis in the hUCMSC-treatment group after transplantation. Vehicle control treated liver showed more serious cellular inflammation and degeneration compared with the hUCMSC treated group (Figure 6(b)).

Serums AST, ALT, MDA, and TBIL levels in injured rat liver increased to 306.28 ± 26.40 U/L, 214.72 ± 23.51 U/L, 10.60 ± 2.46 *μ*mol/L, and 6.97 ± 0.74 *μ*mol/L, respectively, at 24 h after administration of CCl_4_, compared with 138.79 ± 22.45, 63.14 ± 14.24, 5.31 ± 1.61, and 3.47 ± 0.84 in normal rats, confirming acute liver injury by CCl_4_. Mean levels of AST, ALT, and TBIL in the vehicle and model groups were increased at 3 h, 2 days, and 7 days compared with the naive group (Figure 6(a)). Mean levels of MDA and TBIL in the hUCMSC groups were dramatically decreased at 3 h after transplantation, compared with the model and vehicle groups, and recovered to normal levels at 2 days, while mean levels of AST and ALT decreased dramatically at 3 h after transplantation and recovered to normal levels at 7 days (Figure 6(a)). These results indicate that hUCMSCs could promote the recovery of CCl_4_-induced acute liver injury in rats.

## 4. Discussion

In the present study, we used LHS to mimic the liver microenvironment and induce hUCMSCs* in vitro* and showed that hUCMSCs could differentiate into hepatocyte-like cells expressing hepatocyte-specific markers and exhibiting hepatocellular functions. We also transplanted hUCMSCs labeled with PKH26 into rats previously subjected to CCl_4_-induced acute hepatic injury. hUCMSCs were found in the liver of transplanted rats and differentiated into hepatocytes, and the liver structure and function were improved after hUCMSC transplantation. The liver microenvironment is thus a key factor in the differentiation of hUCMSCs into hepatocytes.

The interaction between stem cells and their microenvironment has been considered as the main mechanism regulating stem cell self-renewal and differentiation. Choi et al. found that removal of the pancreas in rats could induce marrow MSCs to differentiate into islet-like cells secreting insulin, glycogen, and somatostatin [15]. We previously induced hUCMSCs to differentiate into nerve-like cells using brain-tissue homogenate supernatant to simulate the microenvironment. LHS may contain many factors and resoluble proteins that could promote the differentiation of hUCMSCs and could mimic the liver microenvironment* in vivo*. After LHS induction, hUCMSCs were found to express the hepatocyte-specific markers AFP, CK18, and TPH2 and to secrete urea and albumin. The treated cells also expressed CYP3A activity, which could metabolize its specific substrate midazolam. Metabolic function is one of the unique features of mature hepatocytes, indicating that the differentiated cells exhibited metabolic function characteristic of liver cells. This effect of induction was superior to that demonstrated in some previous studies [16] and suggested that the liver microenvironment was a significant factor influencing UCMSC differentiation into hepatocytes* in vitro*.

Homing of stem cells is a prerequisite for their therapeutic role. Stem cells injected into veins demonstrated a significant affinity to lesions, and different animal models showed different changes in the microenvironment* in vivo* [17]. Homing therefore appears to be closely related to changes in the microenvironment. The results of our study indicated that hUCMSCs exhibited potent pathotropic migratory properties and became localized to the injured liver. Bayo et al. [18] showed that autocrine motility factors produced by several tumors, including hepatocellular carcinoma, could increase the migration of human mesenchymal stromal cells. Staining of the macrophage marker EMR1 showed that most of the PKH26^+^ cells were active cells, rather than indicating PKH26 that had been taken up by macrophages. Hence, damaged liver might produce factors responsible for enriching hUCMSCs in the liver.

Some studies have found that MSCs can improve liver injury, but whether the mechanism involves a paracrine effect or differentiation remains controversial [19–21]. We found that undifferentiated UCMSCs could express AFP and CK18 and secrete low levels of ALB* in vitro*. This kind of paracrine effect could ameliorate the effects of liver injury on biochemical markers by reducing inflammation. Furthermore, transplanted hUCMSCs differentiated into hepatocyte-like cells in CCl_4_-injured liver tissue and ameliorated the effects on biochemical indicators of liver function and liver tissue structure. These results suggest that paracrine and differentiation effects may both be involved in UCMSC-mediated effects on liver injury at different stages: paracrine effects may ameliorate biochemical markers at the early stage, while differentiation may provide a supportive effect at a later stage after UCMSC transplantation. In addition, differentiated MSCs could continue to secrete useful proteins, thereby maintaining a paracrine effect. However, the ability of hepatocyte-like cells differentiated from MSCs to substitute for necrotic cells and to secrete useful proteins remains crucial, especially in end-stage liver diseases, such as decompensated liver cirrhosis or hepatic failure, where most hepatocytes have lost their function and the replacement effect is thus particularly important. However, it remains unclear if transplanted MSCs will continue to proliferate after differentiation* in vivo*.

## 5. Conclusions

We demonstrated that LHS can partly simulate the hepatic microenvironment* in vivo *and can induce the differentiation of hUCMSCs into functional hepatocytes. hUCMSCs can differentiate into liver cells and exert a therapeutic effect in an acute liver injury microenvironment. However, further studies are needed to investigate the mechanisms in more depth.

## Abbreviations

hUCMSC: Human umbilical cord-derived mesenchymal stem cell
LHS: Liver homogenate supernatant
AFP: *α*-fetoprotein
CK18: Cytokeratin 18
TPH2: Tryptophan 2,3-dioxygenase
ALB: Albumin
ALT: Alanine aminotransferase
AST: Aspartate aminotransferase
MDA: Malondialdehyde
TBIL: Total bilirubin.
